# Association of plasma carnitine levels with bone mineral density and recent osteoporotic fracture

**DOI:** 10.3389/fnut.2025.1664866

**Published:** 2025-11-04

**Authors:** Zhaoyue Shang, Xinwei Wang, Yongliang Du, Xiaohua Zhang, Yanlin Duan, William D. Leslie, Lisa M. Lix, Bo Kan, Shuman Yang

**Affiliations:** ^1^Department of Orthopedics, The First Affiliated Hospital of Jinzhou Medical University, Jinzhou, Liaoning, China; ^2^Department of Epidemiology and Biostatistics, School of Public Health, Jilin University, Changchun, Jilin, China; ^3^Department of Internal Medicine, University of Manitoba, Winnipeg, MB, Canada; ^4^Department of Community Health Sciences, University of Manitoba, Winnipeg, MB, Canada; ^5^Department of Clinical Laboratory, The Second Hospital of Jilin University, Changchun, Jilin, China

**Keywords:** bone health, bone mineral density, carnitine, fracture, metabolomics, osteoporosis

## Abstract

**Background:**

The carnitine system may play an essential role in bone metabolism. However, existing epidemiological studies on the association between carnitine and bone mineral density (BMD) are still controversial. No human study has examined the association of carnitine and osteoporotic fracture. The objective of this research was to examine the association of carnitine levels with BMD and recent osteoporotic fracture.

**Methods:**

We used cross-sectional and case–control studies to examine the associations of carnitine levels with BMD and recent osteoporotic fracture. The cross-sectional study identified 135 participants aged ≥45 years from the Second Hospital of Jilin University. The case–control study identified 44 recent fracture cases and 88 healthy controls aged 50 and older. Multivariable linear regression models were used to test the associations of carnitine with BMD, and conditional logistic regression models were used to analyze the association between carnitine levels and fracture. We used targeted metabolomics technology to measure 27 types of plasma carnitine levels.

**Results:**

In the cross-sectional study, the average age was 57.6 ± 5.0 years, with 29 participants (21.5%) being female. We observed no significant association between total carnitine levels and BMD (*p* > 0.05). In the case–control study, 23 participants (52.3%) were diagnosed with hip fracture. Greater total carnitine levels were negatively associated with the risk of osteoporotic fractures (adjusted odds ratio: 0.43, 95% confidence interval: 0.22–0.85). The magnitude of the associations was comparable for hip and non-hip fractures.

**Conclusion:**

Carnitine was not associated with BMD but was negatively associated with osteoporotic fracture. The low carnitine levels among fracture cases may be due to the post-fracture inflammatory and catabolic stress.

## 1 Introduction

Carnitine (β-hydroxy-y-trimethylammonium butyrate), a conditionally essential nutrient, is present in cells and tissues in the forms of free carnitine and acylcarnitines (1). Carnitine in humans is mainly from meat and dairy products, with a small amount synthesized from lysine and methionine by liver and kidney cells (2). Recently, carnitine deficiency has been reported in conditions such as diabetes, cancer, fatigue, and cardiovascular disease (3–5).

The carnitine system may play an essential role in bone metabolism (6–9). L-carnitine, the biologically active form of carnitine, can reduce bone loss and accelerate fracture healing in ovariectomized rats with osteoporosis (6, 7). Kushwaha et al. (8) suggested that inhibiting fatty acid oxidation *in vivo*, achieved by knocking out carnitine palmitoyl transferase 1a (Cpt1a) in osteoclast precursors, leads to a disruption of osteoclast development in female mice. In human osteoblast-like cells, L-carnitine activates CaMKII and ERKs/AKT signaling cascades to promote cell differentiation and expression of bone matrix proteins (9).

There are limited epidemiological studies investigating the impact of carnitine on BMD in humans (10–12). For example, a cross-sectional study measured serum metabolites in 136 White American women aged 20–40 years old using liquid chromatography-mass spectrometry and found that there was a significant association between isovaleryl carnitine and reduced risk of low BMD (10). Another untargeted metabolomic study on serum samples observed a significant reduction of L-acetylcarnitine and 3-hydroxy-11-octadecenoylcarnitine in the osteoporosis group as compared to the osteopenia group (11). However, to date, no human study has examined the relationship between carnitine and osteoporotic fracture (12).

Therefore, the current research examined the association of carnitine levels with BMD and recent osteoporotic fracture in order to expand our understanding about carnitine and bone health.

## 2 Materials and methods

### 2.1 Study participants

We performed cross-sectional and case–control studies to examine the association of carnitine with BMD and fracture risk, respectively.

#### 2.1.1 Participants’ inclusion and exclusion criteria for the cross-sectional study: association between carnitine and BMD

**Figure 1:**
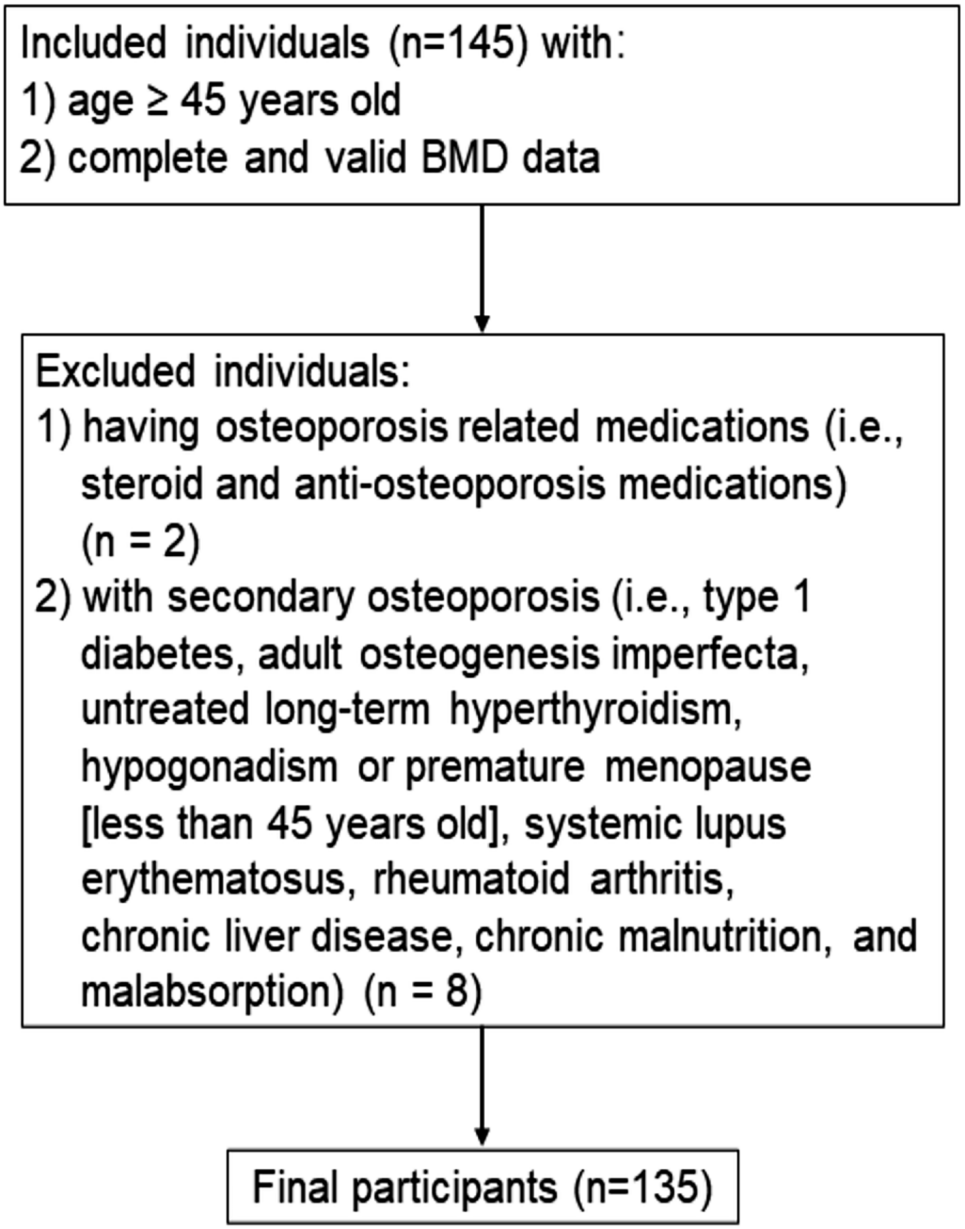
Inclusion and exclusion criteria of the participants in the cross-sectional study.

For the cross-sectional study (Figure 1), participants were identified between June 2019 and September 2019 from the Department of Physical Examination at the Second Hospital of Jilin University in Changchun, Jilin, China. We identified individuals aged ≥45 years with complete and valid data on BMD measurements. We excluded individuals with secondary osteoporosis (i.e., type 1 diabetes, osteogenesis imperfecta, untreated long-term hyperthyroidism, hypogonadism or premature menopause before age 45 years, systemic lupus erythematosus, rheumatoid arthritis, chronic liver disease, chronic malnutrition, and malabsorption). In addition, individuals who were currently using or had previously used relevant bone-active medications (i.e., systemic glucocorticosteroid or anti-osteoporosis medications) were excluded. Finally, a total of 135 participants were enrolled in the cross-sectional study.

All participants signed informed consent forms. The project was approved by the institutional review boards (IRBs) of the School of Public Health, Jilin University (Project #: 2022-02-02), and the Second Hospital of Jilin University (Project #: 2019-13).

#### 2.1.2 Participants’ inclusion and exclusion criteria for the case–control study: association between carnitine and fracture

For the case–control study we used a previously recruited population (13). Inclusion criteria for the case group were individuals aged 50 years or older with newly (1–2 days before enrollment) low-trauma fractures clinically confirmed in the Second Hospital of Jilin University in 2020. All low-trauma fractures were caused by a fall from standing height or lower, low-trauma sports injury or other reasons (i.e., sprain). All fractures including hip, forearm, and humerus fractures were confirmed by x-ray. Controls aged 50 years or older without a history of fracture were identified from the community-based population in the same region. Individuals who were currently using or had previously used relevant bone-active medications (i.e., systemic glucocorticosteroid or anti-osteoporosis medications) were excluded. In addition, we excluded individuals from the case group with pathological fractures and incomplete fracture information; individuals in the control group were excluded if they had secondary osteoporosis.

Cases and controls were matched according to age (±4 years) and sex in a ratio 1:2, respectively. Based on a pilot study, the mean plasma total carnitine level was 24.74 μmol/L in fracture cases and 29.02 μmol/L in controls. To achieve a power of 80% at *α* = 0.05, we estimated that a minimum of 16 cases and 32 controls would be required. All study subjects signed informed consent forms. The project received approval from the institutional review boards (IRBs) of the School of Public Health, Jilin University (Project #: 2022-02-02).

### 2.2 Blood collection

Blood samples were collected after overnight fasting (>8 h of fasting except for water), using heparin anticoagulant tubes (BD, Becton, Dickinson and Company, Franklin Lakes, NJ, USA). In the cross-sectional study, all participants’ blood samples were collected at their first visit before they received any treatment. In the case–control study, blood samples from fracture cases were collected within 2 days of hospital admission, prior to any treatment (such as fracture fixation, hip replacement, or anti-osteoporotic medications). Control participant blood samples were collected during the interview. All blood samples were centrifuged at 1300 × *g* for 10 min at 4 °C to obtain plasma, and stored in a refrigerator at −80 °C until assay.

### 2.3 Carnitine measurement

The plasma samples were thawed at 4 °C before processing and subsequently dropped onto circular filter paper to make small pieces (3 mm in diameter) (14). Metabolite extraction was performed with ethanol and then centrifuged to extract the supernatant. After filtration, the supernatant was transferred to a 96-well plate. Control solutions were composed of carnitine standards (Cambridge Isotope Laboratory, Tewksbury, MA, USA) and quality control products. After the 96-well plate was blown dry with nitrogen, the dry samples in the plate were treated with a mixture of 1-butanol acetyl chloride. Then, the plate was blown dry with nitrogen again. The components to be tested were carried by a mobile phase consisting of 80% acetonitrile aqueous solution and detected by high-performance liquid chromatography-mass spectrometry.

A total of 27 types of carnitines (μmol/L) were measured. Carnitines were classified as free carnitine (C0), short-chain acylcarnitines (SCACs), medium-chain acylcarnitines (MCACs), long-chain acylcarnitines (LCACs), and total carnitines. SCACs included acetylcarnitine (C2), propionylcarnitine (C3), malonylcarnitine (C3DC), butyrylcarnitine (C4), hydroxybutylcarnitine (C4-OH), succinylcarnitine (C4DC), isovalerylcarnitine (C5), hydroxyisovalerylcarnitine (C5-OH), glutarylcarnitine (C5DC), and tiglylcarnitine (C5:1); MCACs are consisted of hexanoylcarnitine (C6), adipylcarnitine (C6DC), octanoylcarnitine (C8), decanoylcarnitine (C10), decenoylcarnitine (C10:1), decadienoylcarnitine (C10:2), and lauroylcarnitine (C12). Myristoylcarnitine (C14), tetradecenoylcarnitine (C14:1), tetradecadienylcarnitine (C14:2), hydroxytetradecanoyl-carnitine (C14-OH), tetradecanoyldiacylcarnitine (C14DC), palmitoylcarnitine (C16), hydroxy-hexadecanoylcarnitine (C16-OH), hydroxypalmitoleoyl-carnitine (C16:1-OH), and stearoylcarnitine (C18) belong to LCACs. Consistent with a previous study (15), total carnitines were the sum of all types of carnitines. Based on the targeted metabolomics platform utilized in our lab, our current detection capability is limited to 27 carnitine species. These species cover four key categories—free, short-chain, medium-chain, and long-chain carnitines—which serve as the basis for quantifying total carnitines. Importantly, similar to previous research (16, 17), the carnitine species we measured are relatively common, hold key roles, and possess clinical relevance as well as importance in metabolic function. We also calculated the ratios of carnitine (e.g., C2 to C0, C3 to C0, and C3 to C2) to determine the impact of specific carnitine catabolism on bone health.

### 2.4 BMD measurement, osteopenia and osteoporosis diagnoses

Lumbar spine (L1–L4) BMD and femoral neck BMD were measured by the Hologic QDR-4500A fan-beam densitometers (Hologic, Bedford, MA) and analyzed by Hologic APEX software (Version 4.0, Hologic, Bedford, MA). According to World Health Organization (WHO) criteria, BMD data were converted to *T*-scores (18). We defined osteoporosis as lumbar spine or femoral neck *T*-score ≤ −2.5 and osteopenia was defined as −2.5 < *T*-score < −1.0 (19).

### 2.5 Ascertainment of covariates

These studies captured information on the following covariates: demographics (age and sex), body mass index (BMI), lifestyle factors (i.e., physical activity, smoking status, frequency of milk intake, and calcium supplement intake), disease history (i.e., coronary heart disease, type 2 diabetes, and stroke), menopausal status, height loss of more than 3 cm after age 40 years, and family history (osteoporosis and fracture). In the case–control study, data on falls or fear of falling due to frailty within the last 12 months were collected. These covariates are established risk factors for the development of fractures and/or osteoporosis (20, 21). We collected histories of fracture from electronic medical records. A standard questionnaire was used by trained staff to collect disease histories from BMD study participants and non-fracture controls through a face-to-face interview. All other information for each participant in both studies was collected through a face-to-face interview. We calculated physical activity levels, measured in metabolic equivalent hours per week (MET-hours/week), based on the frequency and duration of light, moderate, and vigorous physical activities (22). Body weight and height of fracture cases were self-reported, but body weight and height of non-fracture controls and participants in the cross-sectional study were measured directly. BMI was calculated as body weight (kg) divided by body height squared (m^2^).

### 2.6 Statistical analysis

#### 2.6.1 Cross-sectional study: association between carnitine and BMD

The baseline characteristics of study participants were described as means [standard deviations (SDs)] or medians (interquartile ranges) for continuous variables and frequencies (percentages) for categorical variables. Multivariable linear regression models were used to test covariates significantly associated with lumbar spine (L1–L4) and femoral neck BMD (*p* < 0.1). Results are reported as regression coefficients (*β*) and *p* values. We used multivariable linear regression to test the associations of carnitine levels and the carnitine ratio with lumbar spine (L1–L4) and femoral neck BMD. The models were adjusted for all variables significantly associated with BMD at *α* = 0.05; variables selected for inclusion were age, BMI, sex, history of coronary heart disease, history of type 2 diabetes, and height loss >3 cm. Model fit was checked using scatter plots. In the models, if carnitine followed a normal distribution, carnitine was expressed per 1-SD increase. If carnitines were not normally distributed, we transformed and expressed these carnitines per 1-SD increase on the logarithmic scale. To address the issue of multiple testing, we also reported the false discovery rate (FDR; chance of false discovery results) (23). All participants in the cross-sectional study were classified into three subgroups: normal BMD, osteopenia, and osteoporosis. The association between carnitine levels and osteoporosis/osteopenia was then assessed using unconditional multivariable logistic regression models; we adjusted for age, sex, BMI, physical activity, smoking, intake >1 time/week, calcium supplement, history of coronary heart disease, history of type 2 diabetes, history of stroke, height loss >3 cm, family history of osteoporosis, and family history of fractures. The results were reported as adjusted odds ratios (ORs) and 95% confidence intervals (CIs). When analyzing the association of carnitine with osteopenia, osteoporosis cases were excluded from the analysis.

#### 2.6.2 Case–control study: association between carnitine and fracture

Descriptive statistics were conducted for the covariates and carnitine levels by fracture status. Differences between groups on the continuous covariates were compared using Student’s *t*-test or Mann–Whitney U test as appropriate based on the distributional characteristics, while the chi-square test or Fisher exact test for categorical variables. Conditional multivariable logistic regression models were used to analyze the association between carnitine levels and fracture; the models were adjusted for BMI, physical activity, milk intake >1 time/week, and falls of standing height or lower within the past 12 months, which were significantly associated with fracture at alpha = 0.1 in bivariate analysis. Model fit were assessed by examining Nagelkerke *R*^2^ of carnitine associated with fracture (0.796), which indicates a good model fit. Results are reported as adjusted ORs and 95% CIs. In the models, if carnitine followed a normal distribution, the increase in carnitine was expressed per 1-SD increase. Carnitines that were not normally distributed were expressed per 1-SD increase on the logarithmic scale. The FDR was calculated to address the issue of multiple testing.

We also conducted subgroup analyses by fracture site (hip and non-hip). Finally, conditional multivariable logistic regression models were used to analyze the association between the carnitine ratio and fracture; the models were adjusted for BMI, physical activity, milk intake >1 time/week and falls. All the above analyses were performed in the SPSS (version 24.0; SPSS, Chicago, IL) and the R (version 4.3.2; R Foundation for Statistical Computing).

## 3 Results

### 3.1 Cross-sectional study: association between carnitine and BMD

A total of 135 participants were included in the cross-sectional study, with an average age and BMI of 57.6 years and 24.9 kg/m^2^, respectively (Table 1). There were 29 (21.5%) females, among whom the majority were postmenopausal (93.1%). The median physical activity level of participants was 48.1 MET-hours/week. The percentage of participants who smoked and had milk intake >1 time/week was 51.8 and 50.4%, respectively. A minority of participants had a history of coronary heart disease (2.2%), type 2 diabetes (7.4%), stroke (2.2%), and a family history of osteoporosis (4.4%), and fracture (11.1%). There were 63 (46.7%) and 25 (18.5%) participants with osteopenia and osteoporosis, respectively.

**Table 1:** Baseline characteristics of participants in the cross-sectional study.

Characteristic	Mean (SD)/*n* (%)
Age (years)	57.6 (5.0)
Body mass index (kg/m^2^)	24.9 (3.1)
Physical activity (MET-hours/week)	48.1 (35.0, 56.9)
Sex (*n*, %)	
Female	29 (21.5)
Male	106 (78.5)
Smoking (*n*, %)	70 (51.8)
Milk intake >1 time/week (*n*, %)	68 (50.4)
Calcium supplement (*n*, %)	27 (20.0)
History of coronary heart disease (*n*, %)	3 (2.2)
History of type 2 diabetes (*n*, %)	10 (7.4)
History of stroke (*n*, %)	3 (2.2)
Height loss >3 cm (*n*, %)	43 (31.8)
Family history of osteoporosis (*n*, %)	6 (4.4)
Family history of fractures (*n*, %)	15 (11.1)
Lumbar spine BMD *T*-score	−1.1 (1.5)
Femoral neck BMD *T*-score	−0.9 (0.9)
Bone health status (*n*, %)	
Normal	47 (34.8)
Osteopenia	63 (46.7)
Osteoporosis	25 (18.5)

Continuous variables with a normal distribution are presented as means (SDs); continuous variables with a skewed distribution are shown as medians (interquartile ranges); categorical variables are shown as *n* (%).

MET, metabolic equivalent task.

The scatter plots of the relationship between total carnitine and BMD are shown in Supplementary Figure 1. The results of multivariate linear regression models for baseline characteristics and BMD are shown in Supplementary Table 1. A summary of all related supplemental results is shown in Table 2. BMI, history of coronary heart disease and height loss >3 cm was positively associated with lumbar spine BMD, while being female and history of type 2 diabetes were negatively associated with lumbar spine BMD. A statistically significant positive association was observed for BMI and height loss >3 cm with femoral neck BMD, whereas there was a statistically significant negative association of age and female sex with femoral neck BMD.

**Table 2:** Summary of Supplementary materials.

Outcome	Title of Supplementary material
BMD	Supplementary Table 1. Associations of baseline characteristic with lumbar spine BMD and femoral neck BMD
BMD	Supplementary Table 2. Associations of carnitine ratio (per 1-SD increase) with lumbar spine BMD and femoral neck BMD
Fracture	Supplementary Table 3. Carnitine levels of individuals by fracture status
BMD	Supplementary Figure 1. Scatter plots of lumbar spine BMD (A), femoral neck BMD (B) and total carnitine
Osteoporosis	Supplementary Figure 2. Associations between carnitine levels (per 1-SD increase) and osteoporosis
Osteopenia	Supplementary Figure 3. Associations between carnitine levels (per 1-SD increase) and osteopenia
Fracture	Supplementary Figure 4. Associations between carnitine ratio (per 1-SD increase) and fracture

After adjusting for covariates, no statistically significant association was observed between carnitine levels and lumbar spine BMD (all *p* > 0.05; Table 3). However, we observed that total SCACs (*β* = −0.0197, *p* = 0.035) were negatively associated with femoral neck BMD. The FDR for total SCACs was 0.286. In SCACs and MCACs, levels of C2 (*β* = −0.0197, *p* = 0.035) and C8 were negatively associated with femoral neck BMD. After adjusting for all covariates, a statistically significant positive association was observed between the C5DC to C8 ratio and femoral neck BMD (Supplementary Table 2).

**Table 3:** Associations of carnitine levels (per 1-SD increase) with lumbar spine BMD and femoral neck BMD.

Carnitine (μmol/L; abbreviation)	Lumbar spine BMD (g/cm^2^)			Femoral neck BMD (g/cm^2^)		
	*β*	*P*	FDR	*β*	*P*	FDR
Free carnitine (C0)	−0.0121	0.351	0.745	−0.0089	0.342	0.562
Short chain acylcarnitines						
Acetylcarnitine (C2)^a^	−0.0130	0.322	0.745	−0.0199	**0.035**	0.286
Propionylcarnitine (C3)^a^	−0.0167	0.203	0.745	−0.0070	0.455	0.613
Malonylcarnitine (C3DC)^a^	−0.0055	0.680	0.905	−0.0125	0.187	0.446
Butyrylcarnitine (C4)^a^	0.0056	0.687	0.905	−0.0089	0.418	0.589
Hydroxybutyrylcarnitine (C4-OH)^a^	−0.0051	0.696	0.905	−0.0086	0.367	0.562
Succinylcarnitine (C4DC)	0.0012	0.924	0.942	−0.0088	0.336	0.562
Isovalerylcarnitine (C5)	0.0010	0.940	0.942	−0.0173	0.063	0.286
Isovalerylcarnitine (C5-OH)	−0.0125	0.331	0.745	−0.0083	0.381	0.562
Glutarylcarnitine (C5DC)	−0.0034	0.788	0.905	0.0046	0.650	0.746
Tiglylcarnitine (C5:1)	−0.0034	0.788	0.905	−0.0046	0.620	0.746
Total short chain acylcarnitines (SCACs)^a^	−0.0131	0.315	0.745	−0.0197	**0.035**	0.286
Medium chain acylcarnitines						
Hexanoylcarnitine (C6)	−0.0201	0.144	0.745	−0.0169	0.107	0.368
Adipylcarnitine (C6DC)^a^	−0.0038	0.767	0.905	0.0042	0.646	0.746
Octanoylcarnitine (C8)	−0.0224	0.084	0.745	−0.0240	**0.017**	0.286
Decanoylcarnitine (C10)^a^	−0.0162	0.253	0.745	−0.0189	0.070	0.286
Decenoylcarnitine (C10:1)	−0.0023	0.863	0.942	0.0013	0.902	0.911
Decadienoylcarnitine (C10:2)	0.0050	0.703	0.905	0.0086	0.365	0.562
Lauroylcarnitine (C12)^a^	−0.0162	0.206	0.745	−0.0089	0.349	0.562
Total medium chain acylcarnitines (MCACs)	−0.0167	0.218	0.745	−0.0160	0.130	0.368
Long chain acylcarnitines						
Myristoylcarnitine (C14)	0.0067	0.593	0.905	−0.0022	0.813	0.869
Tetradecenoylcarnitine (C14:1)	−0.0177	0.169	0.745	−0.0182	0.074	0.286
Tetradecadienylcarnitine (C14:2)^a^	−0.0131	0.297	0.745	−0.0010	0.911	0.911
Hydroxytetradecanoylcarnitine (C14-OH)^a^	0.0061	0.627	0.905	0.0044	0.629	0.746
Tetradecanoyldiacylcarnitine (C14DC)^a^	−0.0082	0.514	0.905	−0.0143	0.129	0.368
Palmitoylcarnitine (C16)	−0.0119	0.360	0.745	−0.0109	0.229	0.508
Hydroxyhexadecanoylcarnitine (C16-OH)^a^	0.0009	0.942	0.942	0.0035	0.701	0.777
Hydroxypalmitoleoylcarnitine (C16:1-OH)^a^	−0.0065	0.620	0.905	−0.0100	0.302	0.562
Stearoylcarnitine (C18)	−0.0044	0.730	0.905	−0.0133	0.164	0.424
Total long chain acylcarnitines (LCACs)	−0.0137	0.279	0.745	−0.0179	0.066	0.286
Total carnitines	−0.0177	0.173	0.745	−0.0170	0.072	0.286

a Values are per 1-SD increase on the logarithmic scale. Associations were adjusted for age, body mass index, sex, history of coronary heart disease, history of type 2 diabetes, and height loss >3 cm. Bold-faced values indicate statistical significance at *α* = 0.05.

FDR, false discovery rate; *β*, regression coefficients.

After adjusting for all covariates, there was no statistically significant association of total carnitine levels with osteopenia or osteoporosis (Supplementary Figures 2, 3). Among these carnitines, we found that higher levels of C6, C12, and C18 were associated an increased risk of osteopenia. However, the risk of osteoporosis decreased with increasing levels of C10:2 and C14-OH.

### 3.2 Case–control study: association between carnitine and fracture

In the matched case–control study, there were 44 participants in the fracture case group and 88 participants in the non-fracture control group, both with a mean age of 68.2 years. Fractures were attributed to falls, low-trauma sports injuries, and other causes, accounting for 38.6, 38.6, and 22.7% of all fractures, respectively. In the fracture group, 23 participants (52.3%) were diagnosed with hip fracture. Almost all cases were postmenopausal females (96.79%). Compared with the control group, cases had a higher prevalence of falls of standing height or lower within the past 12 months, lower median level of physical activity, and lower percentage of milk intake >1 time/week. Additional descriptive data have been previously published (13).

**Figure 2:**
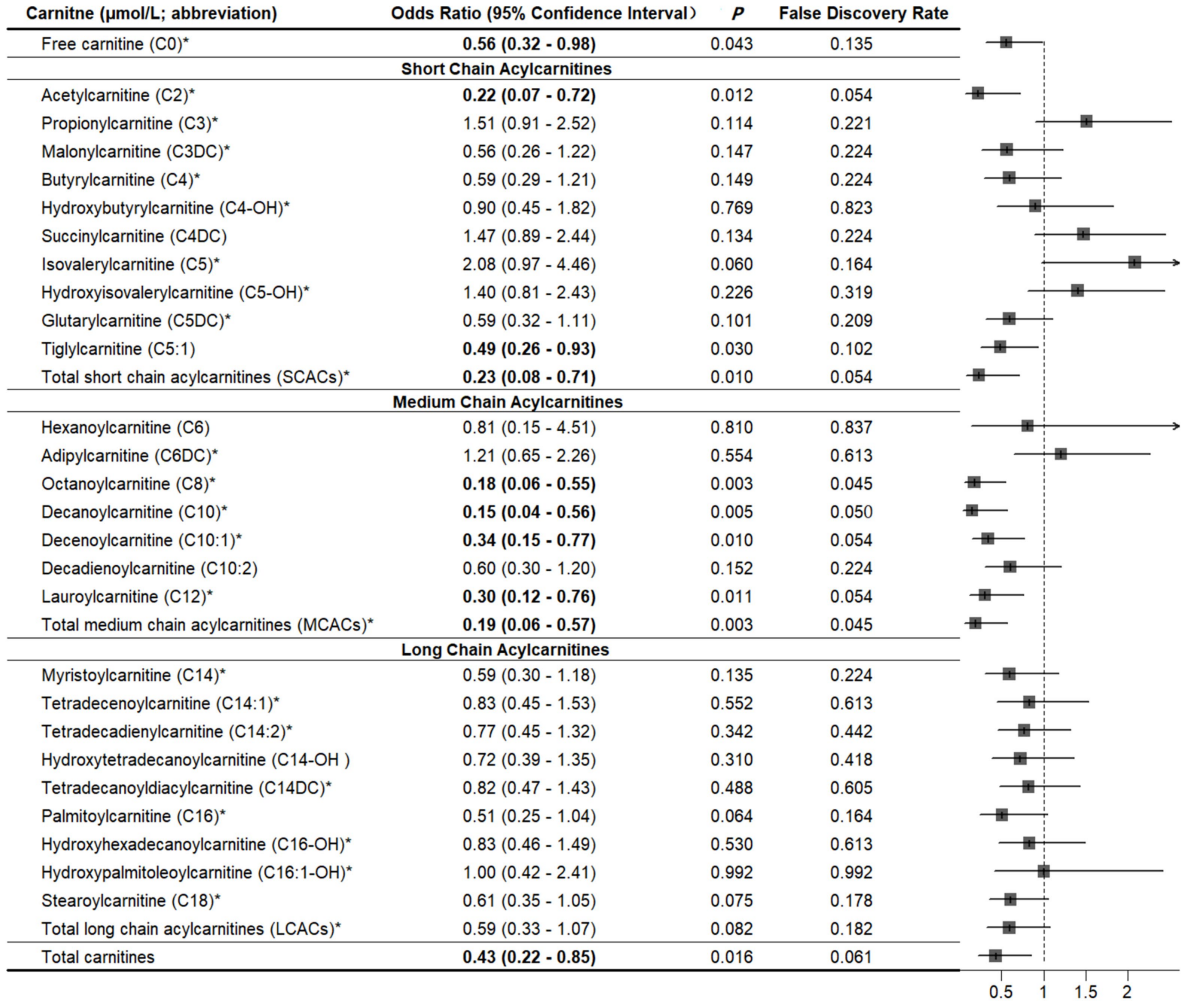
Associations between carnitine levels (per 1-SD increase) and fracture. *Values are per 1-SD increase on the logarithmic scale. Associations were adjusted for body mass index, physical activity, milk intake >1 time/week and falls. Bold-faced values indicate statistically significant at alpha = 0.05.

**Figure 3:**
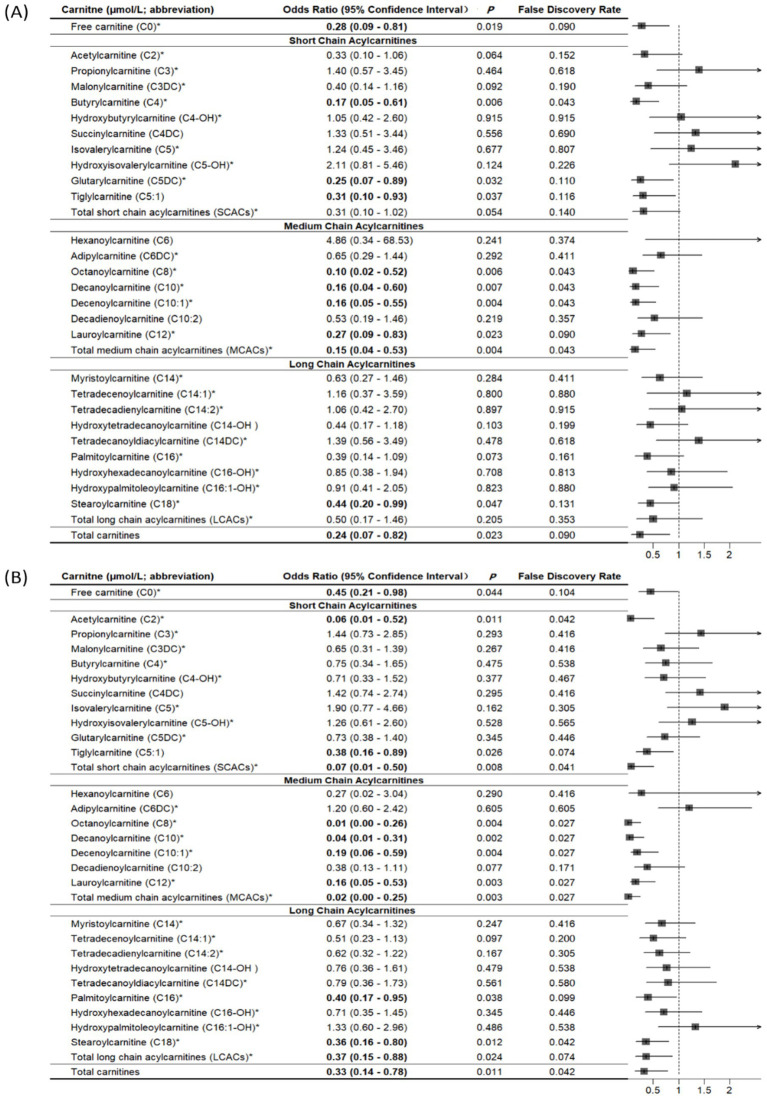
Associations of carnitine levels (per 1-SD increase) with hip fracture **(A)** and non-hip fracture **(B)**. *Values are per 1-SD increase on the logarithmic scale. Associations were adjusted for age, sex, body mass index, physical activity, smoking, milk intake >1 time/week, calcium supplement, history of coronary heart disease, history of type 2 diabetes, history of stroke, height loss >3 cm, falls, family history of osteoporosis, and family history of fracture. Bold-faced values indicate statistically significant at alpha = 0.05.

Compared with controls, cases had significantly lower levels of free carnitine, SCACs, MCACs, LCACs, and total carnitines (all *p* < 0.05; Supplementary Table 3). We summarized all related supplemental results in Table 2. In multivariable conditional logistic regression models adjusted for covariates, increased free carnitine (OR: 0.56, 95% CI: 0.32–0.98), total SCACs (OR: 0.23, 95% CI: 0.08–0.71), total MCACs (OR: 0.19, 95% CI: 0.06–0.57), and total carnitines (OR: 0.43, 95% CI: 0.22–0.85) levels were associated with a reduced risk of fracture (all *p* < 0.05; Figure 2). The FDRs for free carnitine, total SCACs, total MCACs, and total carnitines were 0.135, 0.054, 0.003, and 0.016, respectively. In SCACs, we observed that higher levels of C2 and C5:1 were associated with a lower risk of fracture. In MCACs, levels of C8, C10, C10:1, and C12 were negatively associated with fracture risk. Subgroup analyses by fracture sites suggested that the results of participants with hip or non-hip fractures were consistent with the overall results (Figure 3).

As shown in Supplementary Figure 4, the ratios of C4 to C8, C5 to C0, C5 to C2, C5 to C3, C5-OH to C8, C5-OH to C0, C3DC to C10, and C5DC to C8 were positively associated with fracture risk, while the radios of C5DC to C5-OH and C8 to C2 were negatively associated with fracture risk (all *p* < 0.05).

## 4 Discussion

In the present study, higher total carnitine levels were not associated with lumbar spine and femoral neck BMD, osteopenia, or osteoporosis, but were significantly negatively associated with osteoporotic fracture risk. Among these carnitines, we observed statistically significant negative associations of free carnitine, SCACs (C2, C5:1, and total SCACs), or MCACs (C8, C10, C10:1, C12, and total MCACs) with osteoporotic fracture. The association was comparable for hip and non-hip fractures. In addition, the ratios of C5DC to C5-OH and C8 to C2 were significantly positively associated with fracture, but the ratios of C4 to C8, C5 to C0, C5 to C2, C5 to C3, C5-OH to C8, C5-OH to C0, C3DC to C10, and C5DC to C8 were negatively associated with fracture.

Our cross-sectional study found no statistically significant association of total carnitine levels with BMD, osteopenia or osteoporosis. Similar findings were reported in a 12-week randomized controlled clinical trial of 27 postmenopausal women, in which L-carnitine supplementation had no significant effect on BMD (24). This contrasts with previous studies, in which carnitine derivatives were significantly associated with BMD (10), osteopenia (11), or osteoporosis (25). The reasons for these conflicting results are unclear but may be due to the opposing mechanisms on bone formation (9, 26).

To the best of our knowledge, this is the first study to examine the associations between plasma carnitine levels and osteoporotic fracture. Firstly, lysine and glycine, as precursors and products of the carnitine synthesis pathway, are reduced in fracture patients (27). Secondly, pain and bleeding from the fracture lead to an increased stress state, with activation of inflammatory and catabolic states (28, 29). Under stress conditions, free fatty acids are released by lipolysis from the lipid droplets and transferred into the mitochondria via the palmitoyl-CoA carnitine transferase II shuttle to provide energy to the dying cell (30). This process requires carnitine, which leads to a decrease in plasma total carnitine levels. Finally, another function of carnitine is to ameliorate inflammation by reducing oxidative stress and reactive oxygen species and suppressing lipid peroxidation (31). Thus, fracture patients may have higher consumption of carnitine to maintain redox status and reduce inflammation. Similar findings were reported in an animal study; carnitine treatment promoted callus formation and fracture healing by reducing serum bone turnover markers and pro-inflammatory cytokine levels (6).

We found that ratios of SCACs to C0 (C5 to C0 and C5-OH to C0), SCACs to SCACs (C5 to C2, C5 to C3, and C5DC to C5-OH), SCACs to MCACs (C4 to C8, C5-OH to C8, C3DC to C10, and C5DC to C8) positively associated with fracture. Short- and medium-chain acyls are primarily catalyzed by acyltransferases in peroxisomes and microsomes, whereas long-chain acyls are catalyzed by carnitine palmitoyltransferases I and II on the mitochondrial membrane (32, 33). Therefore, our findings might suggest selective disturbed metabolism of SCACs and MCACs in the peroxisomes and liver microsomes of fracture patients. In this study, the ratios of C5DC to C5-OH and C8 to C2 were negatively associated with fracture risk. Future research is warranted to confirm this.

Compared with the cross-sectional study (45 + years old), we used slightly different age criteria for the case–control study (50 + years old). First, both 45 and 50 years old are used to conduct osteoporosis related studies (34, 35). Second, fracture patients are commonly older than those undergoing BMD screening in the clinical setting. Third, age was adjusted and matched in the BMD and fracture studies, respectively. Using different age cut-offs had little impact on the results or interpretation.

At present, carnitine has been found to have therapeutic potential in various conditions, including type 2 diabetes, myocardial infarction, and kidney disease (36–38). In a rat model of osteoporosis induced by ovariectomy, treatment with L-carnitine—the biologically active form of carnitine—reduced bone loss and accelerated fracture healing, as evidenced by significantly increased callus formation (6, 7). If our findings are confirmed in prospective epidemiologic and interventional studies, carnitine may become a potential target to improve the fracture healing and related outcomes (i.e., re-fracture and mortality). This may be an effective option for patients in the recovery process from fractures.

Our study has several limitations. First, the small sample size may limit the power to identify the associations of carnitine with BMD, osteoporosis, and osteoporotic fractures, increasing the risk of Type II errors. However, our BMD population was larger than a previous study with of 69 participants (11). Second, covariates such as education, occupation, serum calcium, phosphorus and vitamin D levels, calcium intake, and other dietary factors (particularly animal-based foods) were not collected. Potential residual confounding cannot be fully excluded. Third, the reliance on self-reported anthropometric data (weight and height) to calculate BMI in fracture cases introduced potential measurement bias. Fourth, the study population was derived from a single geographic region (Northeast China) and recruited primarily from hospital settings, which constrains external validity and limits generalizability to broader or more diverse populations. Finally, due to the use of cross-sectional and case–control designs, we could not assess temporal associations of carnitine levels with BMD and recent osteoporotic fracture, which limited causal inference.

## 5 Conclusion

Our study found no significant association between carnitine levels and BMD, but carnitine levels were negatively associated with osteoporotic fractures. The low carnitine levels among fracture cases may be due to the post-fracture inflammatory and catabolic stress. During this process, low BMD is not a prerequisite. These findings add to our understanding of the relationship between carnitine and bone health.

## Data Availability

The raw data supporting the conclusions of this article will be made available by the authors, without undue reservation.
