# Supplementary material for: Association of plasma carnitine levels with bone mineral density and recent osteoporotic fracture
Source: Front Nutr. 2025 Nov 4;12:1664866. doi: 10.3389/fnut.2025.1664866 (PMC12623387; doi:10.3389/fnut.2025.1664866)
Supplement: Supplementary file 1 [file Table_1.DOCX]

Supplementary Material

1. **Supplemental Table 1. Associations of baseline characteristic with** **lumbar spine BMD and femoral neck BMD**

| **Characteristic** | **Unit of Change or Comparison** | **Lumbar spine BMD** | | **Femoral neck**  **BMD** | |
| --- | --- | --- | --- | --- | --- |
|  |  | ***β*** | ***P*** | ***β*** | ***P*** |
| Age (years) | 5.0 | -0.0003 | 0.921 | -0.0052 | **0.009** |
| Sex | Female vs. Male | -0.1077 | **0.002** | -0.0954 | **<0.001** |
| Smoking | Yes vs. No | -0.0145 | 0.628 | 0.0114 | 0.621 |
| Physical activity (MET-hours/week) | 1.0 | 0.0003 | 0.733 | 0.0008 | 0.135 |
| Body mass index (kg/m^2^) | 3.0 | 0.0131 | **0.002** | 0.0085 | **0.005** |
| Milk intake >1 time/week | Yes vs. No | 0.0295 | 0.265 | 0.0030 | 0.877 |
| Calcium supplement | Yes vs. No | 0.0011 | 0.972 | 0.0116 | 0.626 |
| History of coronary heart disease | Yes vs. No | 0.2720 | **0.004** | 0.1032 | 0.204 |
| History of type 2 diabetes | Yes vs. No | -0.1912 | **<0.001** | -0.0593 | 0.086 |
| History of stroke | Yes vs. No | -0.0755 | 0.416 | 0.0128 | 0.842 |
| Height loss >3 cm | Yes vs. No | 0.1014 | **0.001** | 0.0467 | **0.025** |
| Family history of osteoporosis | Yes vs. No | -0.0455 | 0.156 | -0.0327 | 0.137 |
| Family history of fractures | Yes vs. No | -0.0002 | 0.992 | -0.0020 | 0.889 |

Bold-faced values are statistically significant at α = 0.1.

Abbreviation: MET: metabolic equivalent task, *β*: regression coefficients.

1. **Supplemental Table 2.** **Associations of carnitine ratio (per 1-*SD* increase) with lumbar spine BMD and femoral neck BMD**

| **Carnitine Ratio (µmol/L; abbreviation)** | **Lumbar spine BMD** | | | **Femoral neck BMD** | | |
| --- | --- | --- | --- | --- | --- | --- |
|  | ***β*** | ***P*** | **FDR** | ***β*** | ***P*** | **FDR** |
| Acetylcarnitine (C2)/Free carnitine (C0)* | -0.0041 | 0.751 | 0.791 | -0.0136 | 0.143 | 0.725 |
| Propionylcarnitine (C3)/Free carnitine (C0)* | -0.0101 | 0.441 | 0.740 | -0.0027 | 0.780 | 0.945 |
| Propionylcarnitine (C3)/Acetylcarnitine (C2)* | -0.0065 | 0.620 | 0.740 | 0.0074 | 0.461 | 0.768 |
| Propionylcarnitine (C3)/Palmitoylcarnitine (C16)* | -0.0052 | 0.700 | 0.777 | 0.0004 | 0.964 | 0.964 |
| Butyrylcarnitine (C4)/Acetylcarnitine (C2)* | 0.0127 | 0.370 | 0.740 | 0.0056 | 0.631 | 0.901 |
| Butyrylcarnitine (C4)/Propionylcarnitine (C3)* | 0.0172 | 0.202 | 0.740 | -0.0015 | 0.879 | 0.964 |
| Butyrylcarnitine (C4)/Octanoylcarnitine (C8)* | 0.0194 | 0.133 | 0.740 | 0.0127 | 0.260 | 0.743 |
| Isovalerylcarnitine (C5)/Free carnitine (C0)* | 0.0073 | 0.566 | 0.740 | -0.0112 | 0.218 | 0.725 |
| Isovalerylcarnitine (C5)/Acetylcarnitine (C2)* | 0.0088 | 0.509 | 0.740 | -0.0008 | 0.932 | 0.964 |
| Isovalerylcarnitine (C5)/Propionylcarnitine (C3)* | 0.0130 | 0.310 | 0.740 | -0.0071 | 0.450 | 0.768 |
| Hydroxyisovalerylcarnitine (C5-OH)/Octanoylcarnitine (C8)* | 0.0096 | 0.448 | 0.740 | 0.0121 | 0.200 | 0.725 |
| Hydroxyisovalerylcarnitine (C5-OH)/Free carnitine (C0)* | 0.0021 | 0.868 | 0.868 | 0.0023 | 0.803 | 0.945 |
| Glutarylcarnitine (C5DC)/Hydroxyisovalerylcarnitine (C5-OH)* | 0.0118 | 0.351 | 0.740 | 0.0153 | 0.112 | 0.725 |
| Glutarylcarnitine (C5DC)/Palmitoylcarnitine (C16)* | 0.0084 | 0.511 | 0.740 | 0.0128 | 0.179 | 0.725 |
| Octanoylcarnitine (C8)/Acetylcarnitine (C2)* | -0.0149 | 0.266 | 0.740 | -0.0094 | 0.350 | 0.768 |
| Octanoylcarnitine (C8)/Decanoylcarnitine (C10)* | -0.0089 | 0.519 | 0.740 | -0.0039 | 0.704 | 0.938 |
| Hydroxyhexadecanoylcarnitine (C16-OH)/Palmitoylcarnitine (C16)* | 0.0062 | 0.629 | 0.740 | 0.0083 | 0.379 | 0.768 |
| Tetradecenoylcarnitine (C14:1)/Palmitoylcarnitine (C16)* | -0.0070 | 0.599 | 0.740 | -0.0064 | 0.518 | 0.797 |
| Malonylcarnitine (C3DC)/Decanoylcarnitine (C10)* | 0.0120 | 0.373 | 0.740 | 0.0080 | 0.413 | 0.768 |
| Glutarylcarnitine (C5DC)/Octanoylcarnitine (C8)* | 0.0238 | 0.064 | 0.740 | 0.0294 | **0.002** | 0.044 |

*Values are per 1-*SD* increase on the logarithmic scale. Associations were adjusted for age, body mass index, sex, history of coronary heart disease, history of type 2 diabetes, and height loss >3 cm. Bold-faced values indicate statistically significant at alpha = 0.05.

Abbreviation: FDR: false discovery rate, *β*: regression coefficients.

1. **Supplemental Table 3.** **Carnitine levels of individuals by fracture status**

| **Carnitine**  **(µmol/L; abbreviation)** | **Fracture Cases**  ***N*=44** | **Controls**  ***N*=88** | ***P*** | **FDR** |
| --- | --- | --- | --- | --- |
| Free carnitine (C0)* | 17.245 (13.417,20.091) | 18.768 (15.955,20.349) | **0.032** | 0.065 |
| **Short Chain Acylcarnitines** | | | | |
| Acetylcarnitine (C2)* | 5.596 (4.247,7.180) | 6.286 (5.523,7.308) | **0.020** | 0.045 |
| Propionylcarnitine (C3)* | 0.238 (0.194,0.331) | 0.313 (0.245,0.382) | **0.001** | 0.006 |
| Malonylcarnitine (C3DC)* | 0.059 (0.034,0.075) | 0.060 (0.043,0.075) | 0.328 | 0.391 |
| Butyrylcarnitine (C4)* | 0.096 (0.074,0.151) | 0.117 (0.090,0.143) | 0.073 | 0.125 |
| Hydroxybutyrylcarnitine  (C4-OH)* | 0.030 (0.016,0.037) | 0.031 (0.016,0.037) | 0.736 | 0.760 |
| Succinylcarnitine (C4DC) | 0.055 (0.022) | 0.050 (0.023) | 0.234 | 0.345 |
| Isovalerylcarnitine (C5)* | 0.098 (0.073,0.110) | 0.088 (0.068,0.112) | 0.272 | 0.363 |
| Hydroxyisovalerylcarnitine (C5-OH)* | 0.045 (0.025,0.057) | 0.038 (0.025,0.051) | 0.296 | 0.367 |
| Glutarylcarnitine (C5DC)* | 0.093 (0.077,0.128) | 0.125 (0.083,0.145) | **0.048** | 0.092 |
| Tiglylcarnitine (C5:1) | 0.022 (0.011) | 0.029 (0.012) | **0.003** | 0.012 |
| Total short chain acylcarnitines (SCACs)* | 6.541 (4.989,8.082) | 7.236 (6.348,8.061) | **0.018** | 0.045 |
| **Medium Chain Acylcarnitines** | | | | |
| Hexanoylcarnitine (C6) | 0.064 (0.026) | 0.060 (0.021) | 0.368 | 0.422 |
| Adipylcarnitine (C6DC)* | 0.026 (0.022,0.029) | 0.024 (0.019,0.033) | 0.826 | 0.826 |
| Octanoylcarnitine (C8)* | 0.081 (0.062,0.115) | 0.141 (0.110,0.168) | **<0.001** | 0.001 |
| Decanoylcarnitine (C10)* | 0.106 (0.079,0.154) | 0.171 (0.129,0.235) | **<0.001** | 0.001 |
| Decenoylcarnitine (C10:1)* | 0.132 (0.096,0.179) | 0.210 (0.156,0.279) | **<0.001** | 0.001 |
| Decadienoylcarnitine (C10:2) | 0.038 (0.018) | 0.051 (0.023) | **0.002** | 0.008 |
| Lauroylcarnitine (C12)* | 0.040 (0.032,0.054) | 0.059 (0.050,0.083) | **<0.001** | 0.001 |
| Total medium chain acylcarnitines (MCACs)* | 0.483 (0.417,0.591) | 0.736 (0.604,0.911) | **<0.001** | 0.001 |
| **Long Chain Acylcarnitines** | | | | |
| Myristoylcarnitine (C14)* | 0.018 (0.016,0.035) | 0.032 (0.017,0.040) | 0.079 | 0.129 |
| Tetradecenoylcarnitine (C14:1)* | 0.059 (0.040,0.091) | 0.071 (0.052,0.091) | 0.274 | 0.363 |
| Tetradecadienylcarnitine (C14:2)* | 0.057 (0.035,0.080) | 0.061 (0.051,0.086) | 0.111 | 0.172 |
| Hydroxytetradecanoylcarnitine (C14-OH ) | 0.022 (0.010) | 0.024 (0.010) | 0.281 | 0.363 |
| Tetradecanoyldiacylcarnitine (C14DC)* | 0.024 (0.021,0.045) | 0.025 (0.022,0.028) | 0.713 | 0.760 |
| Palmitoylcarnitine (C16)* | 0.062 (0.042,0.079) | 0.070 (0.055,0.093) | **0.020** | 0.045 |
| Hydroxyhexadecanoylcarnitine (C16-OH)* | 0.014 (0.013,0.024) | 0.015 (0.013,0.023) | 0.574 | 0.636 |
| Hydroxypalmitoleoylcarnitine (C16:1-OH)* | 0.017 (0.016,0.021) | 0.019 (0.017,0.032) | 0.051 | 0.092 |
| Stearoylcarnitine (C18)* | 0.043 (0.031,0.051) | 0.049 (0.040,0.064) | **0.007** | 0.022 |
| Total long chain acylcarnitines  (LCACs)* | 0.359 (0.279,0.391) | 0.393 (0.332,0.435) | **0.010** | 0.028 |
| Total carnitines | 24.790 (6.053) | 27.434 (5.102) | **0.006** | 0.021 |

Unless otherwise specified, variables with normal distribution are presented as means (SDs); *variables with skewed distribution are shown as medians (interquartile ranges). Bold-faced values indicate statistically significant at alpha = 0.05.

Abbreviation: FDR: false discovery rate.

1. **Supplemental Figure 1. Scatter plots of lumbar spine BMD (A), femoral neck BMD (B) and total carnitine.**

**(A)**


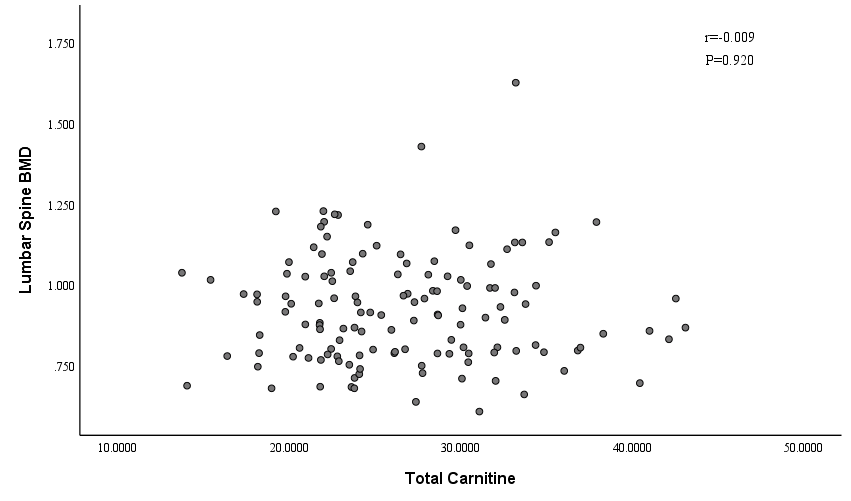


**(B)**


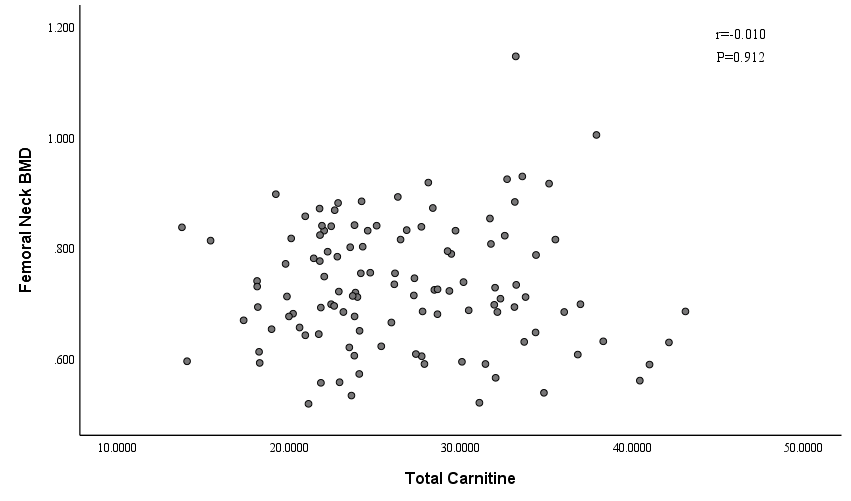


1. **Supplemental Figure 2.** **Associations between carnitine levels (per 1-SD increase) and osteoporosis**


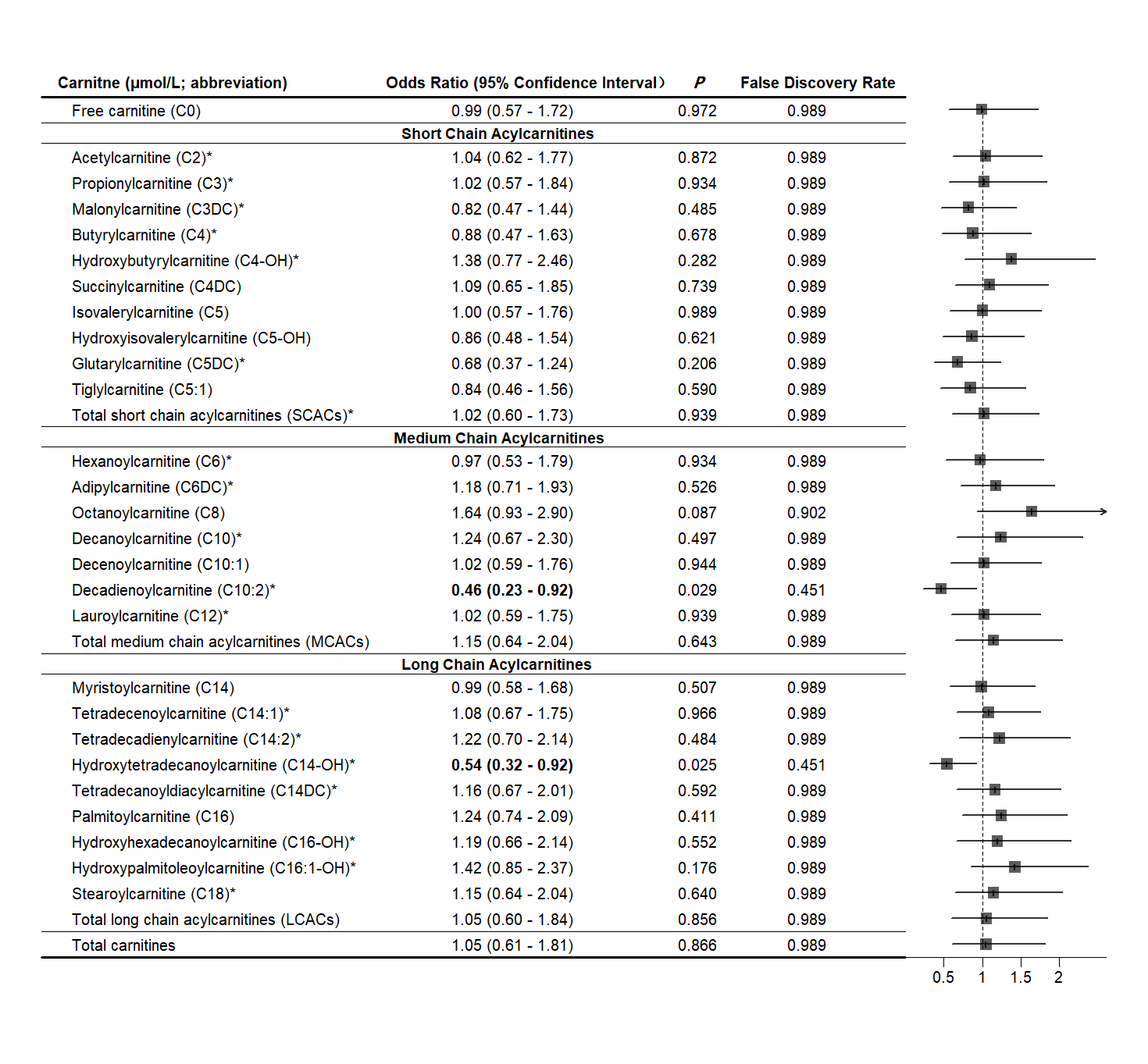


*Values are per 1-SD increase on the logarithmic scale. Associations were adjusted for age, sex, body mass index, physical activity, smoking, milk intake >1 time/week, calcium supplement, history of coronary heart disease, history of type 2 diabetes, history of stroke, height loss >3 cm, family history of osteoporosis, and family history of fracture. Bold-faced values indicate statistically significant at alpha = 0.05.

1. **Supplemental Figure 3. Associations between carnitine levels (per 1-SD increase) and osteopenia**


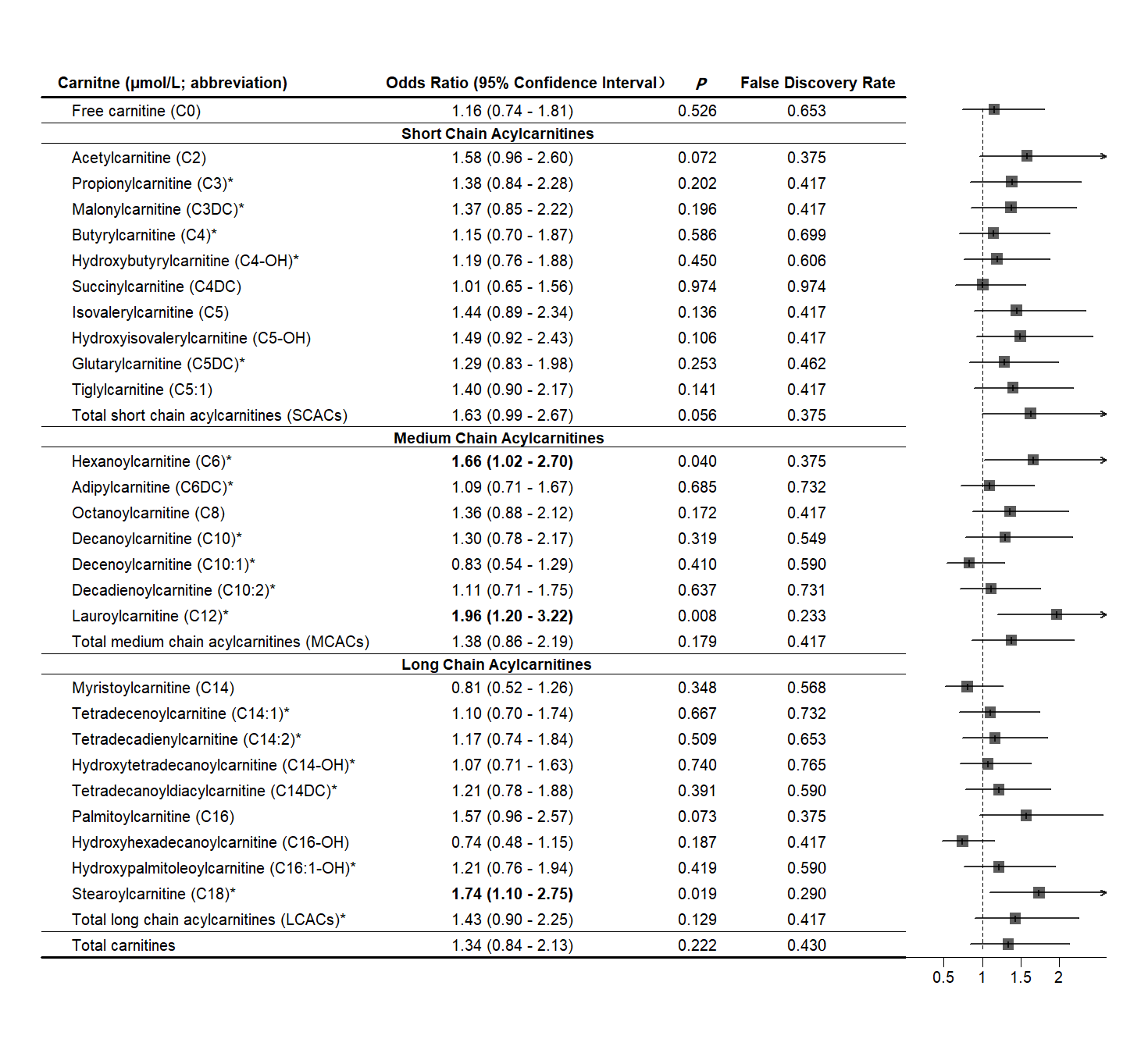


*Values are per 1-SD increase on the logarithmic scale. Associations were adjusted for age, sex, body mass index, physical activity, smoking, milk intake >1 time/week, calcium supplement, history of coronary heart disease, history of type 2 diabetes, history of stroke, height loss >3 cm, family history of osteoporosis, and family history of fracture. Bold-faced values indicate statistically significant at alpha = 0.05.

1. **Supplemental Figure 4. Associations between carnitine ratio (per 1-SD increase) and fracture**


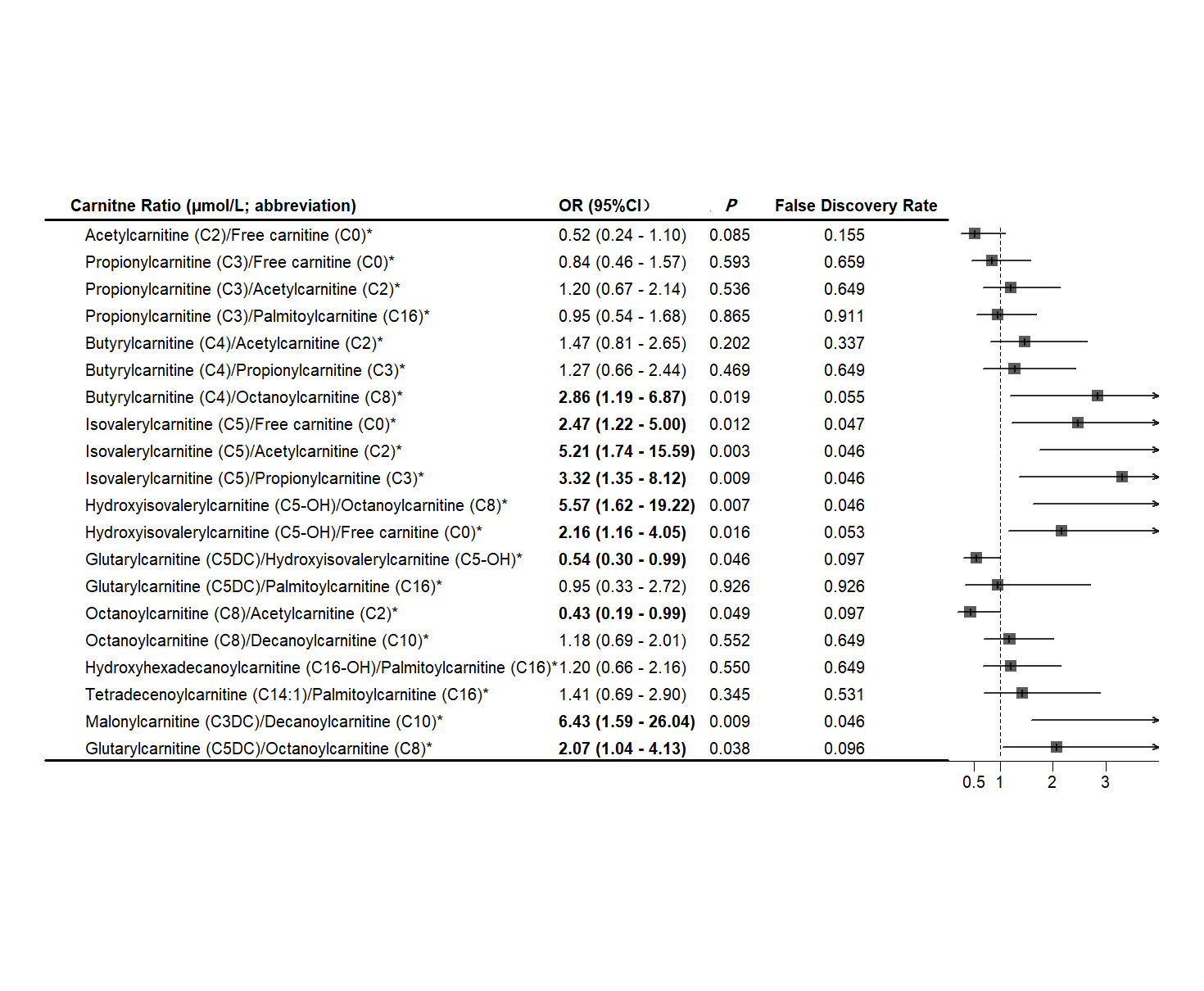


*Values are per 1-SD increase on the logarithmic scale. Associations were adjusted for body mass index, physical activity, milk intake >1 time/week and falls. Bold-faced values indicate statistically significant at alpha = 0.05.

Abbreviation: OR: odds ratio, 95% CI: 95% confidence interval.
